# Assessment of Genetic Diversity and the Population Structure of Species from the *Fusarium fujikuroi* Species Complex Causing *Fusarium* Stalk Rot of Maize

**DOI:** 10.3390/jof10080574

**Published:** 2024-08-14

**Authors:** Prashant P. Jambhulkar, Ruchira Bajpai, Harish Jayarama Reddy, Partha Sarathi Tripathy, Priyanka Varun, Ajaya Kumar Rout, Bijay Kumar Behera, Dilip K. Lakshman, Mallikarjuna Nanjundappa

**Affiliations:** 1 Department of Plant Pathology, College of Agriculture, Rani Lakshmi Bai Central Agricultural University, Jhansi 283004, India; 2 Department of Biotechnology, Noida Institute of Engineering and Technology, Greater Noida 201306, India; 3 College of Fisheries, Rani Lakshmi Bai Central Agricultural University, Jhansi 283004, India; 4 Sustainable Agricultural System Laboratory, USDA-ARS, Beltsville, MD 20705, USA; 5 Zonal Agricultural Research Station, Vishweshwariah Canal Farm, University of Agricultural Sciences, Bangalore 560065, India

**Keywords:** FSR, *Fusarium*, haplotypes, DNA polymorphism, genetic diversity, maize

## Abstract

*Fusarium* stalk rot (FSR), caused by the *Fusarium* species complex, is an economic threat to maize cultivation all over the world. We investigated the population structure and genetic diversity of *Fusarium* species obtained from five major maize-growing regions in India. The *Tef-1α* locus was used for phylogenetic analysis of geographically distinct isolates of *Fusarium verticillioides*, *F. andiyazi*, *F. proliferatum*, *F. nygamai*, and *F. acutatum* causing FSR. A phylogenetic tree showed monophyletic, polyphyletic, and paraphyletic groupings reflecting the complex evolutionary history and genetic diversity within the genus. Monophyletic groupings depicting strong bootstrap support were shown to have a single common ancestor and genetic coherence with limited genetic divergence among sequences. Polyphyletic groupings also presented significant genetic differentiation within the *F. verticillioides* sequences from diverse ecological niches. Nucleotide diversity of moderate level 0.02471 reflected genetic variations within populations that were attributed to factors such as mutation, genetic drift, or varying selection pressures. The Fst value of 0.98205 is particularly indicative of high genetic differentiation, implying that most of the genetic variance is due to differences between populations rather than within them. *F. verticillioides*, with 57 sequences, showed low genetic diversity with three segregating sites and a low haplotype diversity of 0.19486, suggesting the founder effect, where a reduced population expands from a limited genetic pool. The total data estimates across all populations for haplotype analysis showed 72 sequences, 44 segregating sites, and 9 haplotypes with a haplotype diversity of 0.48513. The evolutionary dynamics showed genetic differentiations among *Fusarium* species causing FSR. AMOVA indicated high within-population variations, depicting a substantial genetic diversity within individual populations. The results offer a comprehensive framework for discussing the implications of genetic diversity in pathogen management and the evolutionary dynamics of the *Fusarium* species causing FSR in maize in the Indian subcontinent.

## 1. Introduction

*Fusarium* stalk rot (FSR) in maize is an economically important fungal disease that commonly occurs in 126 maize-growing countries of the world [1,2]. *Fusarium* is a highly pathogenic fungus that impairs plant growth and reduces nutritional value and overall crop yield [3]. Based on its ancestral behavior and shared cultural and morphological traits in common with other *Fusarium* species, FSR is considered to be caused by complex species, including *F. verticillioides* [4,5,6], *F. graminearum* [7,8], *F. falciforme* [9], *F. temperatum*, *F. subglutinans* [10]. In addition to the above reports, four species were reported from maize fields in Mexico [11]. Other countries, including Spain [12], Brazil [13], Canada [14], the USA [15], and India [6], also reported different species as causes of FSR. Nevertheless, *F. verticillioides* was reported in all those countries as being the main species causing FSR in agricultural fields.

FSR is caused by several *Fusarium* species, including members of the *Fusarium fujikuroi* species complex (FFSC). The FFSC is one of the best-studied species complexes, encompassing genera from varied ecologies [16,17]. The FFSC was first established by Wollenweber et al. [18] as a section Liseola for sporodochia-producing species that do not form chlamydospores. *Fusarium* isolates were grouped into three clusters by the proponent of the bio-geographic theory for the FFSC [19], which designated the African, American, and Asian clades based on well-supported phylogenetic groups. The core African clade included maize and coffee pathogens such as *F. verticillioides* and *F. xylarioides* [20,21,22]. Presently, there are more than 60 distinct phylogenetic species recognized under the FFSC [23], *F. verticillioides* is the most predominant species of *Fusarium* in maize-growing areas of India [6,24,25]. *F. verticillioides* is seed-borne, soil or air-borne; however, it may enter plants through wounds, thus making the plant symptomatic and asymptomatic [26]. The initial appearance of FSR occurs at the tassel formation or grain filling stage and attains severity from the milk to waxy stages. Stem bases of susceptible plants become brown with non-distinct spots at the stem. Infected stem tissues become shriveled, loosened, soft, watery, and brown. As the infection spreads, it covers the second and third nodes, and white–pink mycelium appears on the stalk of the plan [27]. In the late stage, plants exhibit symptoms such as drooping, drying, wilting of leaves, empty cob development, and an increased angle between the cob and stalk in the field at later stages of *Fusarium* infection [6]. Other *Fusarium* species are also isolated from infected maize plants exhibiting similar symptoms [28].

The taxonomic identification of *Fusarium* species based on its morphological traits was inefficient with inconclusive species differentiation due to overlapping morphological traits. However, with recent advancements at the molecular level, characterization and identification of species under the FFSC is primarily based on DNA sequence analysis [29]. According to [30], genes of translation elongation factor (*Tef-1α*), calmodulin (*CaM*), and β-tubulin (*Tub2*) could be used for the molecular characterization of most of the *Fusarium* species in the FFSC, while the ITS region, 28 S rDNA and mtSSU genes could not. *Tub2*, *Tef-1α* and RNA polymerase II subunits 1 (*RPB 1*) and 2 (*RPB 2*) are being recommended because these regions can be sequenced easily and can be aligned across the entire genus [28,29,31]. The sequence similarity threshold of *Tef 1-α* is 99.4% and is considered as the most suitable marker to discriminate among *Fusarium* species at the species level. Thus *Tef-1α* is required for reliable identification of unknown isolates of *Fusarium* species for phylogenetic analysis [21]. Genetic diversity and phylogenetic analysis are based on studying the evolutionary relationship of local and global isolates which may or may not correlate with the geographical region or host species. The biological fitness of these genotypes depends on their ability to adapt to changing environmental conditions, thus resulting in a high degree of genetic diversity [32].

The Indian subcontinent is among the most diverse and oldest ecosystems of the subtropical region, and *Fusarium* species in such habitats have evolved with the plant hosts as well as with other pathogens [33]. The objective of the current investigation was to ascertain the genetic and haplotype diversity of specific *Fusarium* species within the FFSC that are responsible for infecting maize crops in India. We employed a phylogenetic methodology to examine and analyze partial sequences of the *Tef-1α* gene and determine the DNA polymorphism of *Tef-1α* sequences of *Fusarium* species isolated from India and the corresponding Gene-Bank reference sequences from other countries. Population genetic studies of pathogenic fungi can provide information on the special distribution of population structure and possible gene flow. In the present study, we determined the haplotype diversity of the *Fusarium* species causing FSR.ITS sequences are less informative and have lower haplotype diversity distribution, resulting in poor resolution and taxa placement in the phylogenetic tree as compared to *Tef-1α* sequences [32]. The global *Tef-1α* sequence datasets of the phylogeny of 11 countries represent the distinct separation of *Incarnatum* and *Equiseti* clades [32]. Haplotype networks help to determine the potential spread of putative pathogens across regions [32,34]. The pathogenicity and aggressiveness of each *Fusarium* species in maize stalks by an artificial inoculation assay were reported in our previous publication [6]. In the present study, the genetic diversity of potentially pathogenic *Fusarium* species among Indian states was assessed. The findings of this study will enhance our understanding of the pathogenicity of different *Fusarium* species, which will be useful in the management of FSR in regard to enhancing maize productivity.

## 2. Materials and Methods

### 2.1. Study Area and Fungal Isolates Sampling

*Fusarium* isolates were obtained from the stalks of naturally infected maize plants showing symptoms of *Fusarium* stalk rot (FSR) between 2020 and 2022. Samples were collected from 40 sites across the southern Rajasthan, eastern Gujarat, western Madhya Pradesh, Karnataka, and Telangana states in India. Geographical locations with coordinates of *Fusarium* species samples are depicted on the map. The locations of the sample collection encompassed five agro-climatic zones of India, namely Zone 7—Eastern Plateau and Hills, Zone 8—Central Plateau and Hills, Zone 9—Western Plateau and Hills, 10—Southern Plateau and Hills, and Zone 13—Gujarat Plains and Hills. Infected plant samples were collected from fields, kept in brown paper bags, brought to the laboratory, and processed for the isolation of pathogens.

### 2.2. Fungal Isolation

The pith of the infected plant samples were surface sterilized by soaking in 0.1% sodium hypochlorite solution for 30 s, washed twice with sterilized distilled water, and plated on potato dextrose agar (PDA) media. These plates were incubated at 27 ± 1 °C in a BOD incubator for 4–5 days. Growing mycelium were moved from PDA to SpeziellerNahrstoffarmer agar (SNA) medium (1 g KH_2_PO_4_, 1 g KNO_3_, 0.5 g MgSO_4_·7H_2_O, 0.5 g KCl, 0.2 g glucose, 0.2 g sucrose, and 20 g agar in 1 L SDW) to enhance sporulation, according to [35]. Single-spore *Fusarium* isolates were obtained using the method described by [36]. Monoconidial cultures grown on SNA media and harvested mycelium and conidia were placed in potato dextrose broth with 15% glycerol and cryopreserved in −80 °C. A total of 74 purified isolates were deposited in the culture collection center, Department of Plant Pathology, Rani Lakshmi Bai Central Agricultural University (RLBCAU), Jhansi, India, for further use in the experiment.

### 2.3. DNA Isolation, PCR Amplification and Sequencing

Freshly sub-cultured *Fusarium* isolates on PDA plates incubated at 27 ± 2 °C in the dark for nearly six to seven days [6] were used for DNA extraction. The genomic DNA was extracted using CTAB method [37]. A 1 cm^2^ plug of fungal mycelium was transferred from the culture plates to a sterile 2 mL Eppendorf tube. Mycelium was macerated using a tissue homogenizer and 500 µL CTAB buffer, then incubated in an Eppendorf tube at 60 °C for 1 h in a hot water bath (Cole-Palmer India Pvt. Ltd., Mumbai, India) with gentle shaking every 10 min. The buffer contained 2.5% CTAB, 4 M NaCl, 20 mM EDTA, 100 mMTris-HCl and 0.2%-Mercaptoethanol; pH 8.0. All buffer preparation components were obtained from Hi-Media^®^ (Thane, Maharashtra, India). The concentration and purity of the DNA was evaluated using Nanodrop (ThermoFisher^TM^ Scientific, Mumbai, India), its absorbance was recorded at 260/280 nm, and the quality of the DNA was tested by running agarose gel (0.8% *w*/*v*) electrophoresis (iGeneLabserve™, Delhi, India). The gel documentation system (Syngene R InGenius3™, Frederick, MD, USA) was used to determine the quality of the DNA. The final DNA volume was adjusted to 50 ng.

The *Tef-1α* gene from each *Fusarium* isolate was amplified using the primer pairs *Tef1α* EF-1 [5′-ATGGGTAAGGA (A/G) GACAEAGAC-3′] and EF-2 [5′-GGA (G/A) GTACCAGT (G/C) ATCATGTT-3′] [31]. The PCR reaction was performed in a 50 µL reaction volume containing 1 ng DNA template, 1.5 mM MgCl_2_, 0.5 mM each of dNTP, 0.4 µm of forward and reverse primers, and 1.25U of Taq DNA polymerase. The PCR amplification was performed with an initial denaturation at 94 °C for 5 min followed by 35 cycles each at 94 °C for 1 min, annealing at 50 °C for 1 min, extension at 72 °C for 2 min, and a final extension at 72 °C for 10 min in a Thermocycler (Veriti^TM^, Applied Biosystem^TM^, New Delhi, India). The PCR products were separated by agarose gel electrophoresis (1% *w*/*v* in 0.5 × TAE buffer) and visualized by ethidium bromide staining. The PCR products were sequenced through the Sanger sequencing platform at Medauxin™, Bangalore, India. The sequences received were edited using BioEdit software v7. 0.9 [38] and compared with sequences in NCBI (National Center for Biotechnology Information) using nBLAST software (version +2.15.0). The sequences were deposited in NCBI GenBank and the accession numbers are listed in Table S1.

### 2.4. Phylogenetic Analysis

Phylogenetic relationships among the *Fusarium* sequences were analyzed using MEGA11 software Version 11 [39]. The sequences were aligned using the MUSCLE algorithm [40] within MEGA11 to ensure accurate alignment of the nucleotide sequences. The phylogenetic tree was constructed using the neighbor-joining (NJ) method, which is effective for reconstructing phylogenies from distance data. To assess the robustness of the phylogenetic tree, a bootstrap analysis was performed with 10,000 replicates. This method provides a measure of confidence for the inferred tree topology by repeatedly resampling the data and recalculating the tree. Nodes with high bootstrap values indicate strong support for the corresponding clades in the phylogenetic tree. The final phylogenetic tree was visualized and edited in MEGA11, with bootstrap values displayed at the corresponding nodes to indicate the level of support for each clade. An outgroup, LR583646.1 *Nectria haematococca*, was included to root the tree and provide context for the relationships among the *Fusarium* sequences. The maximum likelihood tree was inferred using MEGA11 after finding the best fit DNA model. Models with the lowest BIC scores (Bayesian Information Criterion) are considered to describe the substitution pattern the best.

### 2.5. DnaSP Analysis

DNA polymorphism was analyzed using DnaSP v6.12.03 software [41]. The analysis included 74 *Fusarium* sequences, covering a region from position 1 to 720. Two types of analyses were performed: pairwise comparisons and individual site analyses. Gaps or missing information were excluded only in pairwise comparisons. This approach resulted in an average of 509.38 sites analyzed across 2701 pairwise comparisons. In the individual site analysis, 628 sites were analyzed, identifying polymorphic sites and calculating average differences, nucleotide diversity (Pi), and Theta-W values per sequence and per site. The population analysis included four distinct populations, analyzing a region from position 1 to 720, with a total of 720 sites. Sites with alignment gaps were excluded, resulting in 363 sites analyzed. The populations were designated as FV, FAC, FAN, and FP. For each population, the number of sequences, segregating sites, haplotypes, haplotype diversity (Hd), average number of differences (K), nucleotide diversity (Pi), and nucleotide diversity with Jukes and Cantor correction (PiJC) were calculated. Genetic differentiation among the populations was evaluated using Fst and Nm [42].

### 2.6. Haplotype Network Analysis

The sequences were edited to equal size using BioEdit software and then aligned using the MUSCLE algorithm inside MEGA11. The alignment file was imported into DnaSP for haplotype analysis. The DnaSP algorithm then proceeded to identify unique haplotypes based on variations such as single-nucleotide polymorphisms (SNPs), insertions, and deletions. We carefully adjusted the settings within DnaSP to handle indels and missing data appropriately, ensuring that the haplotype delineation was both accurate and reflective of the underlying genetic diversity. The output from DnaSP provided us with a comprehensive list of haplotypes, their frequencies, and a matrix of haplotype differences. For the construction and visualization of the haplotype network, we used PopART v1.7 [43]. Importing the haplotype data from DnaSP into PopART, we opted for the Median-Joining (MJ) network algorithm due to its efficiency in handling our dataset complexity and its ability to accurately depict mutational relationships between haplotypes. We adjusted the epsilon value to zero, adhering to the recommended settings for constructing a network that closely reflects the genetic distances and connections among our identified haplotypes. The visualization in PopART allowed us to represent haplotypes in a manner that highlighted their frequencies and the mutational steps between them, using size and lines, respectively. We further enriched our network visualization by incorporating geographical information and color coding to represent different populations, thereby adding layers of interpretative value to our genetic data.

### 2.7. AMOVA Analysis

The genetic data were analyzed using the software package Arlequin v3.5.2.2. The analysis of molecular variance (AMOVA) was performed to partition the genetic variation within and among populations. The AMOVA was based on the allele frequencies at the 537 loci, and the calculations were weighted by the number of loci to obtain an average estimate. The sum of squares, variance components, and percentage of variation were calculated for each source of variation (among populations and within populations). The total genetic variance was partitioned to determine the proportion of variation attributable to differences among populations and within populations. The Fst value, a measure of population differentiation, was calculated from the AMOVA results. The Fst value was determined by dividing the variance component among populations by the total variance component.

### 2.8. Statistical Analysis

All statistical analyses were performed using Arlequin software, which is specifically designed for population genetics data. Descriptive statistics, including the sum of squares and variance components, were generated and summarized. The percentage of variation for each source of variation was calculated to understand the distribution of genetic diversity.

## 3. Results

### 3.1. Collection of Isolates

**Figure 1:**
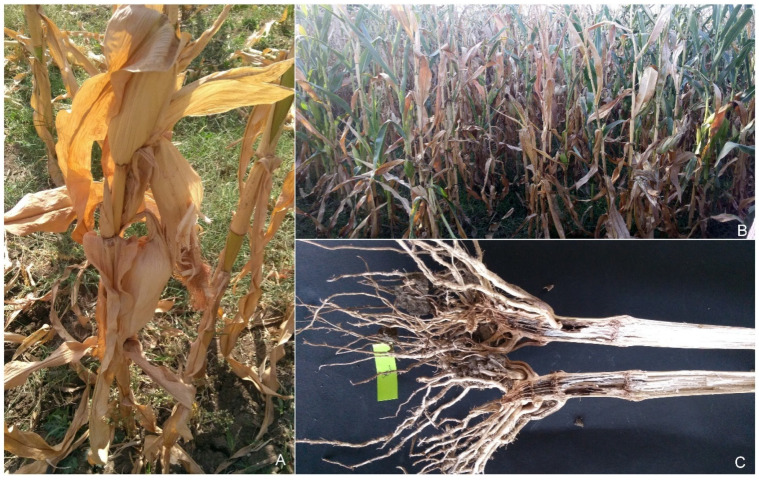
Disease symptoms in FSR-affected maize plant; (**A**): drooping, wilting, and drying of leaves, empty cob development, and an increase in the angle between stalks and cobs in the field; (**B**): severely affected field with *Fusarium* stalk rot; (**C**): vascular discoloration of the infected stem.

**Figure 2:**
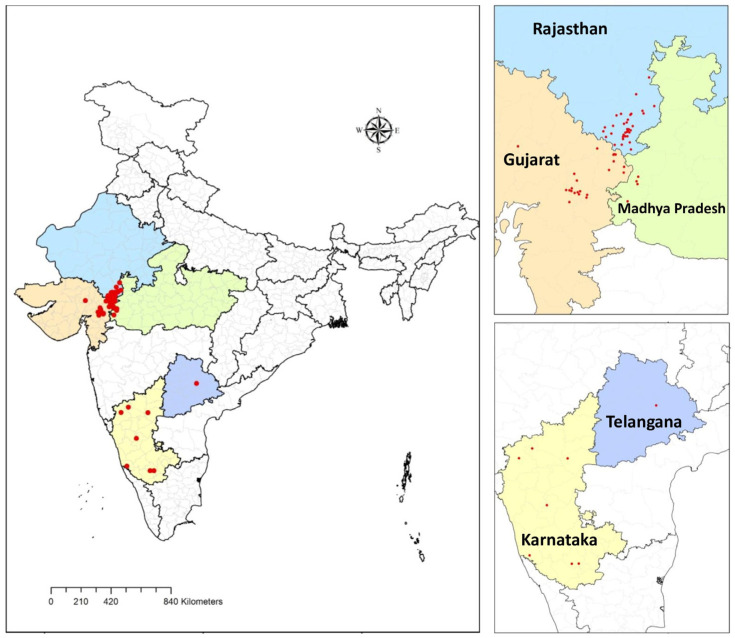
Geographical distribution of 74 strains isolated from maize-growing states of India with FSR incidence. Arc GIS 10.1 platform (ESRI Inc., Redlands, CA, USA) was used for preparing the map for the study area and the sample location sites, with inputs of the geographic co-ordinates of 74 sites.

**Figure 3:**
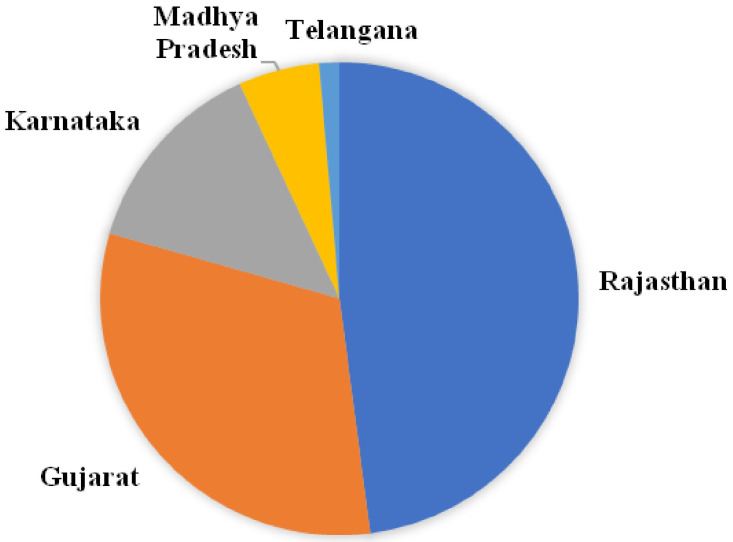
Isolation frequencies of number of isolates collected from different states of India.

In the present study, we selected maize-growing regions of India with high incidence of FSR. Diseased plants exhibiting symptoms such as drooping, drying, wilting of leaves, and empty cobs were selected to collect samples (Figure 1). Seventy-four isolates were collected from 40 sites. These sites include 16 regions of Southern Rajasthan, 4 sites of Madhya Pradesh, 5 regions of Gujarat, 7 regions of Karnataka, and 1 site from Telangana State (Figure 2). The isolation frequencies varied between the five states surveyed for sampling: Rajasthan (49.2%), Gujarat (32%) and Madhya Pradesh, Karnataka, and Telangana (together contributing 20% of the isolates in the study) (Figure 3). From each region, we selected two–three fields having maize plants with typical FSR symptoms to collect diseased samples.

### 3.2. Molecular Identification

Approximately 600 bp segments were amplified for the *Tef-1α* region. Sequences of *Tef-1α* loci of *Fusarium* species were evaluated in combination with other sequences of the FFSC available on the NCBI GenBank database. The sequences identity of *Tef-1α* fragments of 74 strains of *Fusarium* species belonging to the FFSC was about 85% (pairwise identity ~95%). The sequences identified in the present study have been deposited in the NCBI GenBank and the accession numbers are present in Table S1.

### 3.3. Phylogenetic Analysis and Evolutionary Relationship of Fusarium Strains Causing FSR

The nucleotide sequences of 74 representative isolates of *Fusarium* species varied from 538 to 831 bp and shared ≥90% nucleotide sequence similarity with *Tef-1α* sequences of *Fusarium* species available in the GenBank. Approximately 700–720 bp segments were amplified for the *Tef-1α* region. The gene sequences of 74 *Fusarium* species isolates were used to construct a phylogenetic tree using the neighbor-joining method and maximum likelihood method.

**Figure 4:**
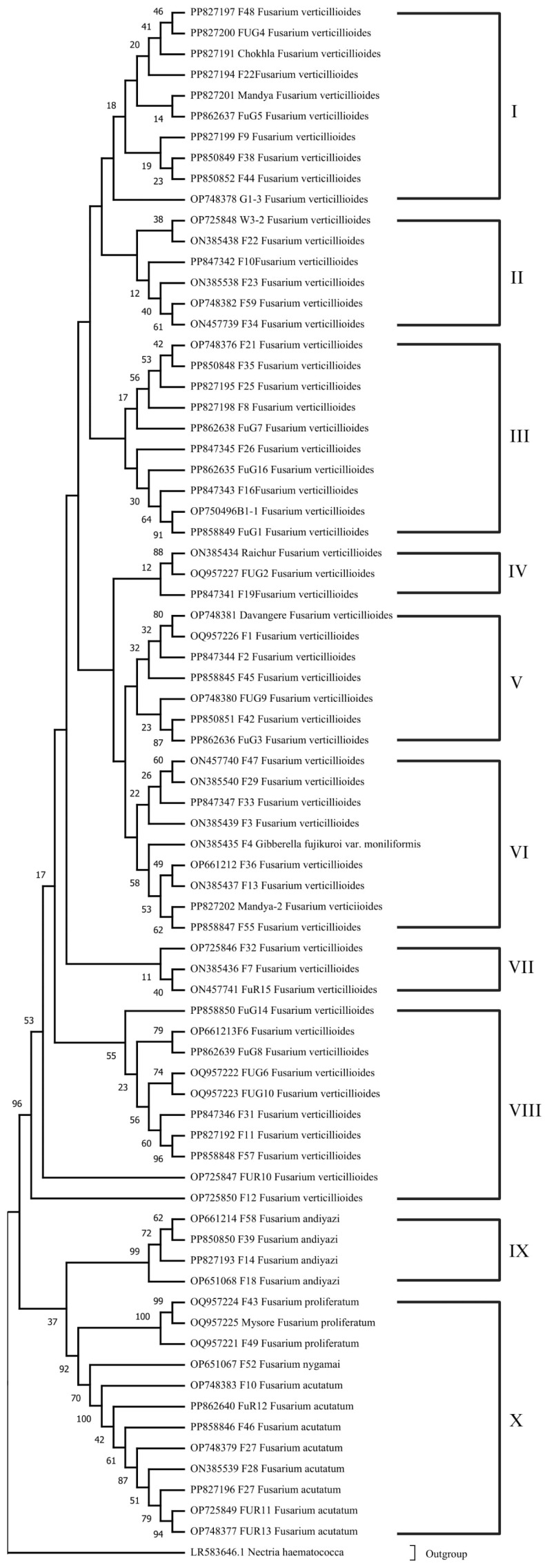
Phylogenetic tree constructed by a *Tef-1α* gene sequence using the neighbor-joining (NJ) tree method. Bootstrap values are indicated above nodes based on 10,000 replicates of the data. The Romar numerals (I–X) are depicting individual clusters of *Fusarium* spp. strains.

The phylogenetic relationships among the *Fusarium* sequences were analyzed using MEGA11. The phylogenetic tree, constructed based on the sequences, revealed distinct clades representing different *Fusarium* species (Figure 4). The analysis included a variety of *Fusarium verticillioides* sequences from various geographical locations and hosts. It was noticed that all the 74 *Fusarium* species strains were grouped into seven major clusters: Cluster I (10 isolates), Cluster II (six isolates), Cluster III (10 isolates), Cluster IV (three isolates), cluster V (seven isolates), Cluster VI (nine isolates), Cluster VII (three isolates), Cluster VIII (10 isolates), Cluster IX (four isolates) and Cluster X (12 isolates). Cluster I has six isolates from Gujarat, three from Rajasthan, and one from Karnataka that were identified as *F. verticillioides* and which were grouped together on the basis of genetic similarity. Cluster II showed close similarity with 38% bootstrap value between the W3-2 isolate from Telangana and the F22 isolate from Rajasthan. The other four isolates are from Gujarat and Rajasthan. Cluster III surprisingly showed a close similarity (91% bootstrap value) between the B1-1 isolate from Karnataka and the FuG1 isolate from Gujarat. Four isolates, F21, F35, F25, and F8, from Rajasthan had bootstrap values between 42 and 56% which were distantly correlated with an isolate from Gujarat FuG7 with a 17% bootstrap value. Similar to Cluster III, Cluster IV also showed a high bootstrap value of 88% to show close similarity between the Karnataka isolate and the Raichur and Gujarat isolate FUG2. Cluster V showed a high bootstrap value of 87% between the F42 MP isolate and the FUG3 Gujarat isolate. Similarly, the Davanagere, Karnataka isolates and the F1 Rajasthan isolate have a closeness with 80% bootstrap value. Cluster VI is segregated mainly in two sub-clusters. The first sub-cluster clubbed three Gujarat isolates with one Rajasthan isolate of *F. verticillioides* with bootstrap values ranging between 22 and 60%. A second sub-cluster with 49–62% bootstrap values clubbed isolates from Madhya Pradesh, Gujarat, Rajasthan and Karnataka. Cluster VII clubbed three Rajasthan *F. verticillioides* isolates. Cluster VIII had F11 and F57 *Fusarium verticillioides* isolates with 96% bootstrap values, which were in close vicinity, while FUG14, an isolate from Gujarat had a 55% bootstrap vale with most of the isolates (F6, FUG8, FUG6, FUG10) of the cluster. Cluster IX, with four isolates grouped together, belongs to the *F. andiyazi* species from Rajasthan and Gujarat. Cluster X having three *F. proliferatum* isolates with high bootstrap values (99–100%). It also clubbed *Fusarium acutatum* isolates with minimal similarity with *F. verticillioides* isolates with a boot strap value of 37%.

Overall, several well-supported clades were observed in the phylogenetic tree. *Fusarium verticillioides* sequences clustered together indicated genetic similarities among these sequences. Notably, sequences labeled PP827197 F48, PP827200 FUG4, and PP827191 Chokhla *Fusarium* verticillioides formed a strongly supported clade with a bootstrap value of 46. Similarly, other *Fusarium verticillioides* sequences, such as PP827194 F22 and PP827201 Mandya *Fusarium verticillioides*, also formed distinct clades with high bootstrap values. Additionally, sequences from other *Fusarium* species, including *Fusarium proliferatum* and *Fusarium acutatum*, were also present in the phylogenetic tree. *Fusarium proliferatum* sequences, such as OQ957224 F43 and OQ957225 Mysore *Fusarium proliferatum*, were clustered together with high bootstrap support of 100. Similarly, *Fusarium acutatum* sequences, including OP651067 F52 and OP748383 F10, formed a well-supported clade with a bootstrap value of 100. The phylogenetic tree also included an outgroup, represented by LR583646.1 *Nectria haematococca*, which helped to root the tree and provide context for the relationships among the *Fusarium* sequences.

**Figure 5:**
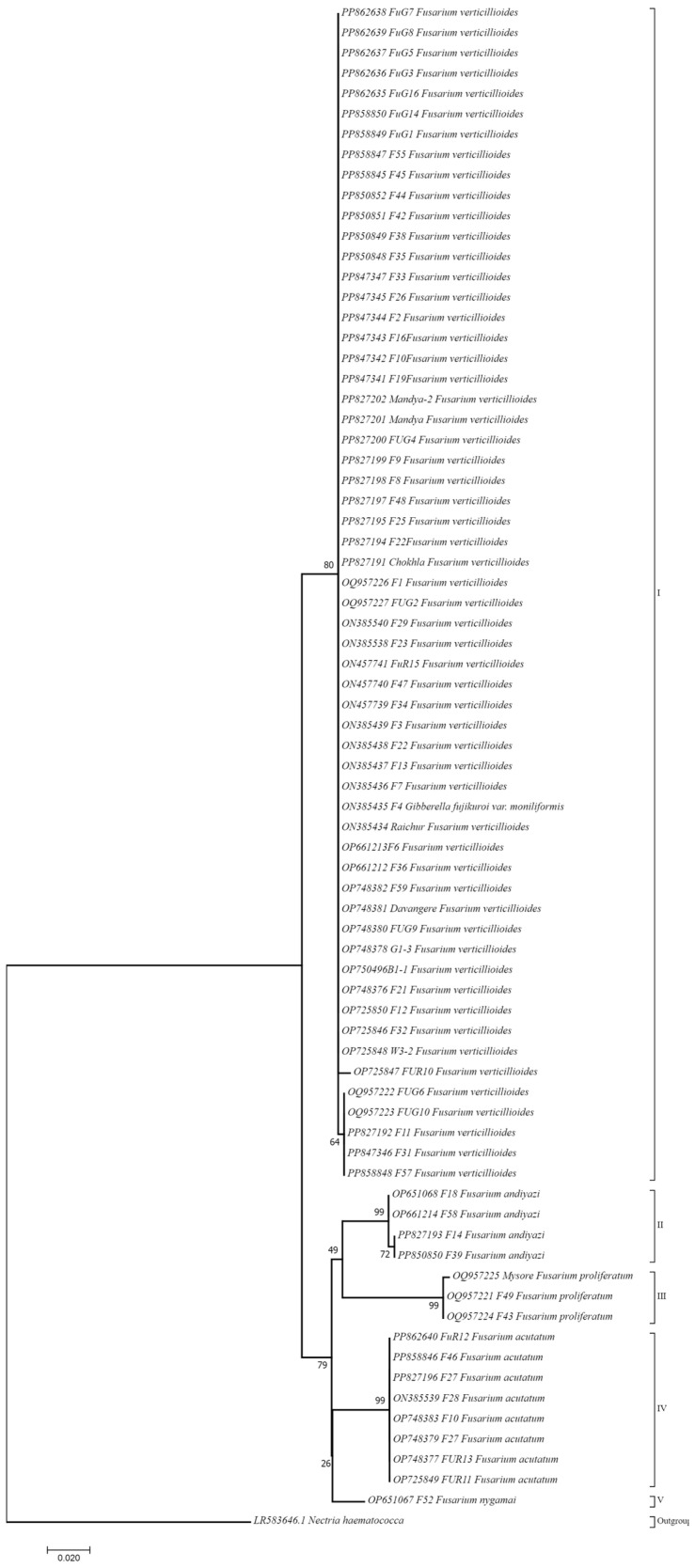
Phylogenetic tree constructed by the *Tef-1α* gene sequence using the maximum likelihood analysis of a *Fusarium fujikuroi* species complex. The outgroup includes *Nectria haematococca*. The percentage of trees in which the associated taxa are clustered together has been shown next to the branches. The scale bar displays the expected number of nucleotide substitutions per site. The Roman numerals (I–V) are depicting clusters of *Fusarium* spp. strains.

The best-fit DNA model for the ML tree was analyzed using MEGA11. The ML tree demonstrated convoluted and nested structures of the FFSC and contained *F. verticillioides*, *F. acutatum*, *F. proliferatum*, *F. nygamai*, and *F. andiyazi* (Figure 5). Models with the lowest BIC scores (Bayesian Information Criterion) are considered to describe the substitution pattern the best. K2 + G model was found to have the lowest BIC, i.e., 3592.764. For estimating ML values, a tree topology was automatically computed. The analysis involved 75 nucleotide sequences. The codon positions included were first + second + thirrd + Noncoding. All positions containing gaps and missing data were eliminated. There was a total of 360 positions in the final dataset. Evolutionary analyses were conducted in MEGA11. The evolutionary history was inferred by using the maximum likelihood method based on the Kimura 2-parameter model. The tree with the highest log likelihood (−1036.21) has been shown. Initial tree(s) for the heuristic search were obtained automatically by applying neighbor-joining and BioNJ algorithms to a matrix of pairwise distances estimated using the Maximum Composite Likelihood (MCL) approach and then selecting the topology with a superior log likelihood value. A discrete Gamma distribution was used to model evolutionary rate differences among sites (five categories (+G, parameter = 0.5662)). The tree is drawn to scale, with branch lengths measured in the number of substitutions per site. The analysis involved 75 nucleotide sequences. The codon positions included were first + second + third + noncoding. All positions containing gaps and missing data were eliminated. There was a total of 360 positions in the final dataset.

### 3.4. DNA Polymorphism and Genetic Differentiation Analysis

The analysis of DNA polymorphism was conducted on 74 *Fusarium* sequences across a region spanning from 1 to 720, comprising a total of 720 sites (Table 1). After excluding sites with alignment gaps or missing data, 363 sites were analyzed. For pairwise comparisons, where gaps or missing information were excluded only in pairwise comparisons, an average of 509.38 sites were analyzed across 2701 pairwise comparisons. This resulted in an average of 12.544 differences and a nucleotide diversity (Pi) of 0.02471. The individual site analysis involved 628 sites, with 109 being polymorphic. The average number of differences was 17.202, yielding a nucleotide diversity (Pi) of 0.02739. Additionally, the Theta-W values per sequence and per site were 24.10441 and 0.03838, respectively.

The population analysis included four populations within the same selected region, excluding sites with alignment gaps to retain 363 sites (Table 2). Population *F. verticillioides* (FV) consisted of 57 sequences, featuring three segregating sites, three haplotypes, a haplotype diversity (Hd) of 0.19486, an average of 0.23308 differences, and a nucleotide diversity (Pi) of 0.00064 (PiJC: 0.00064). Population *F. acutatum* (FAC), with eight sequences, showed no segregating sites or haplotypes, resulting in a haplotype diversity (Hd) of 0.0, an average of 0.0 differences, and a nucleotide diversity (Pi) of 0.0 (PiJC: 0.0). Population *F. andiyazi* (FAN), comprising four sequences, had one segregating site and two haplotypes, with a haplotype diversity (Hd) of 0.66667, an average of 0.66667 differences, and a nucleotide diversity (Pi) of 0.00184 (PiJC: 0.00184). Population *F. proliferatum* (FP), with three sequences, had one segregating site and two haplotypes, with a haplotype diversity (Hd) of 0.66667, an average of 0.66667 differences, and a nucleotide diversity (Pi) of 0.00184 (PiJC: 0.00184). The total data estimates for all populations combined included 72 sequences, 44 segregating sites, nine haplotypes, a haplotype diversity (Hd) of 0.48513, an average number of nucleotide differences (Kt) of 7.64358, and a nucleotide diversity (PiT) of 0.02106.

The genetic differentiation among the populations was assessed, yielding the following key parameters: Hs: 0.19871, Hst: 0.59040, Ks: 0.24934, Kst: 0.96738, Ks *: 0.15074, Kst *: 0.87486, Z: 818.96190, Z *: 6.64954, and Snn [44]: 1.00000. Gene flow estimates included Fst: 0.98205, Nm: 0 (Table 3).

### 3.5. Haplotype Network Analysis Results

**Figure 6:**
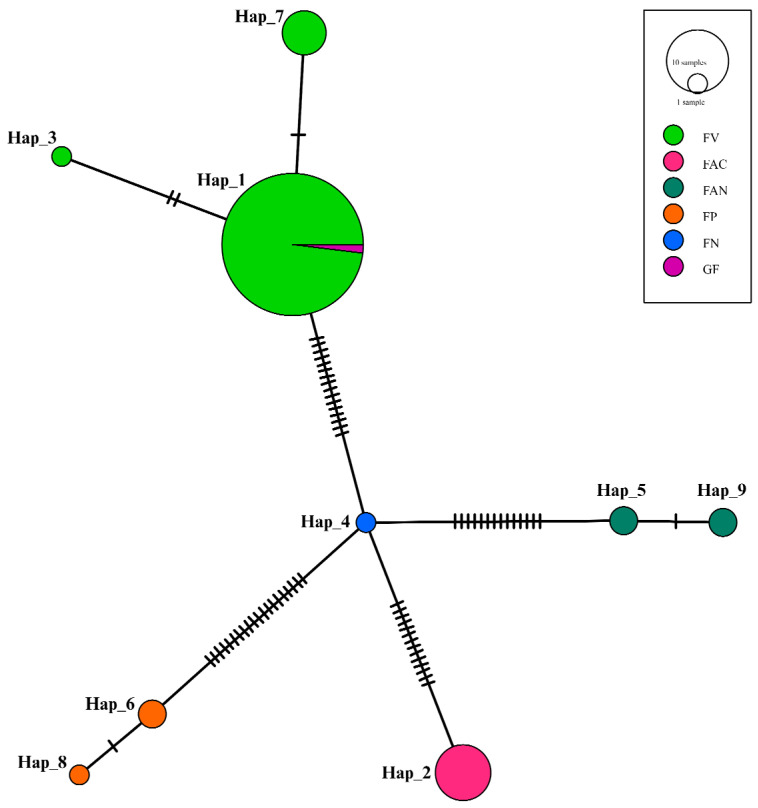
A haplotype network derived from concatenated sequencing data of the Tef 1-α gene was constructed for 74 *Fusarium* species isolates collected from the pith of the infected stem of maize plants. All haplotypes were interconnected with identified single-nucleotide polymorphisms. Each haplotype (denoted as Hap) was analysed by inter-island composition to identify possible spread. *Fusarium verticillioides* had the most haplotypes that indicated possible spread, likely because FSR was a target for the survey with multiple symptomatic maize plants. Black lines on the branches are used to indicate the number of mutational changes between two different haplotypes.

The haplotype network analysis was performed to visualize the genetic relationships among different haplotypes of *Fusarium* species (Figure 6). The network showed nine distinct haplotypes (Hap_1 to Hap_9), represented by circles whose sizes were proportional to the number of samples for each haplotype. Different populations from which the haplotypes were derived includes, i.e., *F. verticillioides* (FV), *F. accutatum* (FAC), *F. andiyazi* (FAN), *F. proliferatum* (FP), *F. nygamai* (FN), and *Gibberella fujikuroi* (GF). The network structure showed that Hap_1 is the most prevalent haplotype, predominantly composed of samples from the FV population, with GF inside it. This indicates a high degree of genetic homogeneity within the FV population and suggests that Hap_1 is a common ancestral haplotype or has a high reproductive success. Hap_4 presented as a central node connecting several other haplotypes, indicating it might be an intermediate or ancestral haplotype from which other haplotypes have diverged. Notably, Hap_4 includes samples from the FN population, suggesting it plays a crucial role in linking various genetic lineages. Hap_2 was composed entirely of samples from the FAC population, showing a distinct genetic lineage separate from the FV and FN populations. This distinct separation suggests limited gene flow between the FAC population and other populations, possibly due to geographical or ecological barriers. The network also showed several smaller haplotypes (Hap_3, Hap_5, Hap_6, Hap_7, Hap_8, and Hap_9) connected to the central haplotypes by multiple mutation steps. These haplotypes were less frequent and were specific to certain populations, indicating recent divergence or rare genetic variants within these populations.

### 3.6. AMOVA Analysis

**Figure 7:**
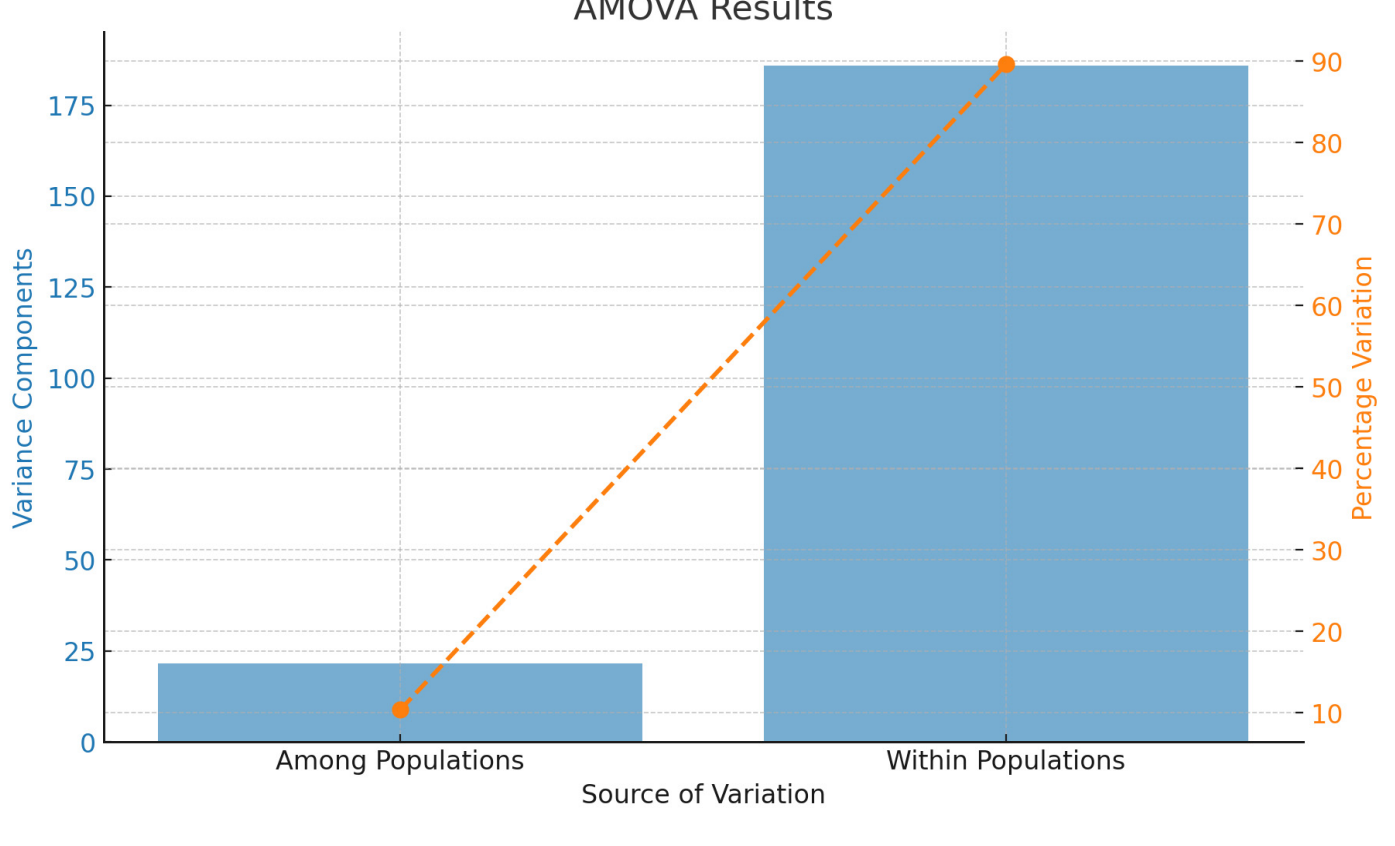
AMOVA results showing variance components and percentage of variation. The bar plot illustrates the variance components attributable to genetic variation among populations (21.51) and within populations (185.85). The line plot with markers represents the percentage of total genetic variation accounted for by among-population differences (10.37%) and within-population differences (89.63%). The figure highlights that most of the genetic variation is found within populations.

These results summarize the AMOVA conducted over 537 loci, highlighting the contributions of variation among and within populations to the total genetic variance. The total sum of squares was calculated to be 28,560.293. Among populations, the sum of squares was 2170.955, resulting in a variance component of 21.50947, which accounts for 10.37% of the total genetic variation. Within populations, the sum of squares was 26,389.338, with a variance component of 185.84532, accounting for 89.63% of the total genetic variation. The total variance component across all sources of variation was 207.35479 (Table 4). The analysis shows that 10.37% of the total genetic variation is attributable to differences among populations, while 89.63% is attributable to differences within populations (Figure 7). The Fst value, which measures the proportion of genetic variance that can be attributed to population differentiation, was calculated as follows:

Fst = Variance Among Populations Total Variance = 21.51/207.35479 = 0.1037

## 4. Discussion

The phylogenetic analysis of the *Fusarium* sequences, represented in the constructed neighbor-joining (NJ) tree, reveals evolutionary relationships and significant genetic diversity among the different *Fusarium* species. The use of 10,000 bootstrap replicates ensures robust and reliable support for the inferred phylogenetic relationships, providing a clear depiction of the evolutionary history and genetic structure of the *Fusarium* genus.

The tree exhibits both monophyletic and polyphyletic groupings, highlighting the complexity of genetic relationships within this genus. Monophyly is observed in several clades, where sequences from the same species cluster together, supported by high bootstrap values [45]. The clade containing sequences PP827197 (F48), PP827200 (FUG4), and PP827191 (Chokhla) *F. verticillioides* showed strong bootstrap support, indicating a single common ancestor and genetic coherence within this group. This monophyletic clustering suggests limited genetic divergence and possible recent common ancestry, which could be due to geographical proximity or host specificity [46].

In contrast, polyphyletic groupings were also observed, where sequences from the same species do not form a single clade but are dispersed across different branches of the tree [45]. This is exemplified by the distribution of *F. verticillioides* sequences in multiple distinct clades. Such polyphyletic patterns indicate significant genetic differentiation within the species, potentially resulting from diverse ecological niches, host associations, or evolutionary genetic exchange with other *Fusarium* species. This has earlier been studied in other species [47,48,49]. The polyphyletic nature of these groupings underscores the genetic complexity and evolutionary dynamics at play within *F. verticillioides*.

Paraphyly was also observed in some instances, where a single clade contains a common ancestor but does not include all descendant species [50]. This can be seen in clades where *F. verticillioides* sequences cluster together but exclude other closely related species. This paraphyletic grouping suggests that, while there is a common evolutionary lineage, not all descendants are represented within the same clade, indicating possible lineage-specific evolution or selective pressures, leading to divergence. This is also evident from earlier works [51,52].

The presence of well-supported clades with high bootstrap values, such as the 100% bootstrap support for *F. proliferatum* sequences OQ957224 (F43) and OQ957225 (Mysore), highlights the robustness of the phylogenetic inference and confirms distinct genetic lineages within the genus. These findings are crucial for understanding the genetic basis of pathogenicity, host range, and ecological adaptation in *Fusarium* species.

The ML tree in Figure 5 could resolve the phylogenetic relationships among identified species in this species complex. The obtained species-specific clades showed closely related species clustered together.

Overall, the phylogenetic tree constructed using MEGA11 showed the observed monophyletic, polyphyletic, and paraphyletic groupings, reflecting the complex evolutionary history and genetic diversity within the genus. These insights are essential for advancing our understanding of *Fusarium* biology, informing disease management strategies, and guiding future research on fungal genetics and evolution.

The DNA polymorphism analysis using DNASP v6.12.03 through *Tef-1α* sequences has provided a comprehensive insight into the genetic diversity present within sequences of *Fusarium* species from diverse geographical regions in India. The results showed that out of the 720 sites analyzed, 363 sites were retained after excluding those with alignment gaps or missing data, highlighting the importance of data quality in genetic studies. The pairwise comparisons revealed an average of 509.38 sites analyzed per comparison, with an average of 12.544 differences being observed. The nucleotide diversity (Pi) was calculated to be 0.02471, which reflects the degree of genetic variation present within the population. This relatively moderate level of nucleotide diversity indicates some genetic variability among the sequences, which could be attributed to factors such as mutation, genetic drift, or varying selection pressures. This is also evident from earlier works by various researchers [53,54,55]. The analysis of individual sites provided a more detailed view, with 628 sites being analyzed and 109 being identified as polymorphic. The average number of differences at individual sites was higher at 17.202, resulting in a slightly higher nucleotide diversity (Pi) of 0.02739. The Theta-W values per sequence and per site were 24.10441 and 0.03838, respectively, suggesting a higher rate of polymorphism and potential population expansion or long-term stable population size [56,57].

The population analysis revealed distinct differences in genetic diversity across the four populations studied. Population FV, with 57 sequences, showed low genetic diversity with only three segregating sites and a haplotype diversity (Hd) of 0.19486. This low diversity might suggest a recent bottleneck or founder effect, where the population size was significantly reduced and then expanded from a limited genetic pool. Similar results were also found in the earlier studies on the fungal population [58,59]. In contrast, Population FAC exhibited no segregating sites and a haplotype diversity of 0, indicating no genetic variation within this group. This could be due to a very recent common ancestor or strong selective pressures maintaining a uniform genetic makeup. Populations FAN and FP showed higher genetic diversity, with one segregating site each and a haplotype diversity of 0.66667. The nucleotide diversity (Pi) for both populations was 0.00184, which is low but higher than that of Population FV, suggesting some degree of genetic differentiation and potential historical recombination events. The effect of recombination on the fungal population has also been studied in earlier works [60,61]. The total data estimates across all populations showed 72 sequences, 44 segregating sites, and nine haplotypes with a haplotype diversity of 0.48513. The overall nucleotide diversity (PiT) was 0.02106, indicating moderate genetic variability across the entire dataset.

The genetic differentiation analysis provided insights into the evolutionary dynamics between the populations. The Gst value of 0.55137 and DeltaSt value of 0.02014 indicate moderate to high genetic differentiation among populations. High values of GammaSt (0.96981) and Nst (0.98276) further support the presence of substantial genetic structure and limited gene flow between populations. The Fst value of 0.98205 is particularly indicative of high genetic differentiation, implying that most of the genetic variance is due to differences between populations rather than within them. The genetic differentiation results highlight the complex evolutionary processes shaping the genetic structure of *Fusarium* populations. Factors such as geographic isolation, ecological specialization, and historical population dynamics likely play critical roles in driving genetic divergence and maintaining distinct genetic lineages within the *Fusarium* genus. These findings underscore the importance of considering genetic differentiation in the management and control of *Fusarium*-related plant diseases, as different populations may exhibit varying pathogenicity and resistance profiles.

The haplotype network analysis of *Fusarium* species showed the genetic structure and evolutionary dynamics within and between different populations. The network’s structure, characterized by distinct haplotypes with varying frequencies, underscores the complexity of *Fusarium* genetic diversity and suggests that several evolutionary processes are at play. This diversity is crucial for the resilience and adaptability of the species, enabling populations to withstand environmental changes and selective pressures [48]. The observed haplotype diversity indicates that the *Fusarium* populations we studied are genetically diverse, with nine distinct haplotypes identified from a relatively large dataset of 74 sequences having wider geographical distances. This level of diversity is significant, suggesting that these populations can rapidly adapt to environmental changes and potentially overcome disease management strategies that are not based on a comprehensive understanding of the pathogen’s genetic makeup.

The predominance of Hap_1, mainly composed of samples from the FV population, suggests a common ancestral haplotype or one with a high reproductive success. This high frequency could be attributed to selective advantages conferred by Hap_1, allowing it to dominate the FV population. Such patterns are often observed in populations where specific haplotypes confer adaptive benefits, leading to their prevalence over others [62]. The presence of GF within Hap_1 indicates some level of gene flow or shared ancestry between these populations. It is also evident from earlier works that *G. fujikuroi* is also another name for *F. verticillioides*, as it releases Gibberellin [63]. Hap_4’s role as a central node connecting multiple haplotypes highlights its potential significance as an intermediate or ancestral haplotype. This central position suggests that Hap_4 might represent a genetic bridge facilitating the divergence of other haplotypes through mutation or recombination events. The inclusion of FN samples within Hap_4 emphasizes its importance in linking various genetic lineages and suggests historical gene flow between FN and other populations. The distinct clustering of Hap_2, which consists solely of FAC samples, indicates limited genetic exchange between the FAC population and others. This genetic isolation could result from geographical barriers, ecological niches, or reproductive isolation mechanisms preventing gene flow [64]. Such patterns of genetic isolation are critical for understanding the speciation processes and the maintenance of genetic diversity within populations.

The presence of several smaller haplotypes (Hap_3, Hap_5, Hap_6, Hap_7, Hap_8, and Hap_9) connected to the central haplotypes by multiple mutation steps suggests recent divergence events or rare genetic variants. These smaller haplotypes are often indicative of recent mutations or genetic drift within populations, reflecting ongoing evolutionary processes [65]. The population-specific nature of these haplotypes highlights localized evolutionary pressures or historical demographic events shaping the genetic landscape. The observed genetic structure within the haplotype network suggests a combination of historical gene flow, genetic drift, and selection pressures shaping *Fusarium* populations. The limited gene flow between certain populations, such as FAC and others, highlights the importance of barriers to genetic exchange in maintaining genetic diversity and promoting divergence. Understanding these barriers is crucial for managing *Fusarium*-related diseases, as genetic diversity within and between populations can influence pathogen virulence, host specificity, and resistance to control measures.

The results of the AMOVA indicate that there is significant genetic differentiation among the populations studied. The among-population variance component accounted for 10.37% of the total genetic variance, with the remaining 89.63% being attributable to within-population variation. This pattern of genetic structure is consistent with other studies on similar taxa, where within-population variation often exceeds among-population variation [66,67].

The calculated Fst value of 0.1037 further confirms the moderate level of genetic differentiation among populations. According to Wright’s, 1980 [68] guidelines, an Fst value between 0.05 and 0.15 indicates moderate genetic differentiation. This suggests that, while there is some genetic exchange between populations, barriers to gene flow exist, possibly due to geographical, ecological, or behavioral factors [69].

The high within-population variation observed in this study is indicative of substantial genetic diversity within individual populations. This diversity can be attributed to various factors, including large effective population sizes, gene flow, and local adaptation. It is crucial for the adaptive potential of populations, allowing them to respond to environmental changes and reducing the risk of inbreeding depression [66,70]. Comparatively, the among-population variation, though smaller, is significant and suggests that populations are not completely homogeneous. This could be due to the presence of local adaptations or historical events that have shaped the genetic structure of these populations.

## 5. Conclusions

The comprehensive molecular analyses provide essential insights into the genetic diversity, evolutionary history, and population structure of *Fusarium* species. This study provides a foundation for future research on the evolutionary biology of *Fusarium* species and underscores the importance of considering genetic diversity in the management of *Fusarium* diseases. It is imperative for disease management approaches to incorporate strategies that address the genetic variability and potential for adaptation in the pathogen. Integrated disease management strategies, including crop rotation, resistant cultivars, and fungicide applications, should be designed with an understanding of the genetic landscape of the pathogen populations to enhance their efficacy and sustainability.

Furthermore, our findings highlight the utility of genetic markers and advanced statistical analyses in unravelling the complex genetic relationships among pathogen populations. Such insights are invaluable for the development of targeted and sustainable disease management strategies that can adapt to the dynamic nature of pathogen populations. In summary, the genetic differentiations among *Fusarium* species populations revealed in this study contribute to a deeper understanding of the genetic structure and evolutionary dynamics of these pathogens. This knowledge is crucial for managing the *Fusarium* stalk rot of maize and for further research into the genetic mechanisms underlying the adaptability and pathogenicity of *Fusarium* species.

## Figures and Tables

**Table 1:** DNA polymorphism analysis. This table presents the results of DNA polymorphism analysis conducted on *Fusarium* sequences. The analysis includes the number of sites analyzed, the number of polymorphic sites (S), the average number of differences, nucleotide diversity (Pi), and Theta-W values per sequence and per site for both pairwise comparisons and individual site analysis.

Analysis Type	Number of Sites Analyzed	Number of Polymorphic Sites, S	Average Number of Differences	Nucleotide Diversity, Pi	Theta-W, Per Sequence	Theta-W, Per Site
Pairwise Comparisons	509.38 (average)	N/A	12.544	0.02471	N/A	N/A
Individual Sites (column by column)	628.00	109	17.202	0.02739	24.10441	0.03838

**Table 2:** Population analysis. This table summarizes the population analysis of *Fusarium* sequences across four populations (FV, FAC, FAN, and FP). It includes the number of sequences, the number of segregating sites (S), the number of haplotypes (h), the haplotype diversity (Hd), the average number of differences (K), and the nucleotide diversity (Pi) with and without Jukes and Cantor correction (PiJC).

Population	Number of Sequences	Number of Segregating Sites, S	Number of Haplotypes, h	Haplotype Diversity, Hd	Average Number of Differences, K	Nucleotide Diversity, Pi	Nucleotide Diversity with JC, PiJC
FV	57	3	3	0.19486	0.23308	0.00064	0.00064
FAC	8	0	1	0.0	0.0	0.0	0.0
FAN	4	1	2	0.66667	0.66667	0.00184	0.00184
FP	3	1	2	0.66667	0.66667	0.00184	0.00184
Total Data Estimates	72	44	8	0.48513	7.64358	0.02106	N/A

**Table 3:** Genetic differentiation. This table provides the genetic differentiation estimates between different *Fusarium* populations. It includes parameters such as Hs, Ks, Kxy, Gst, DeltaSt, GammaSt, Nst, Fst, Dxy, and Da, highlighting the genetic structure and differentiation among the populations FV, FAC, FAN, and FP.

Population 1	Population 2	Hs	Ks	Kxy	Gst	DeltaSt	GammaSt	Nst	Fst	Dxy	Da
FV	FAC	0.17570	0.20440	19.12281	0.53712	0.01130	0.95334	0.99411	0.99391	0.05268	0.05236
FV	FAN	0.21142	0.26151	19.62281	0.26440	0.00650	0.90534	0.97787	0.97707	0.05406	0.05282
FV	FP	0.20329	0.25476	26.45029	0.24162	0.00683	0.91187	0.98380	0.98299	0.07287	0.07163
FAC	FAN	0.16667	0.22222	17.50000	0.57333	0.02112	0.97872	0.98154	0.98095	0.04821	0.04729
FAC	FP	0.09524	0.18182	25.33333	0.59648	0.02744	0.98798	0.98744	0.98684	0.06979	0.06887
FAN	FP	0.66667	0.66667	22.83333	0.19864	0.03017	0.95833	0.97199	0.97080	0.06290	0.06107

**Table 4:** Global AMOVA results (weighted average over 537 loci) as analyzed from Arlequin v3.5.2.2.

Source of Variation	Sum of Squares	Variance Components	Percentage Variation
Among populations	2170.96	21.51	10.37
Within populations	26,389.34	185.85	89.63
**Total**	28,560.29	207.35	100.00

## Data Availability

Data are contained within the article and Supplementary Materials.

## Supplementary Materials

The following supplementary information can be downloaded at: https://www.mdpi.com/article/10.3390/jof10080574/s1. Table S1: Accession numbers of *Fusarium* species strains deposited in NCBI GenBank.
