# Supplementary material for: Assessment of Genetic Diversity and the Population Structure of Species from the Fusarium fujikuroi Species Complex Causing Fusarium Stalk Rot of Maize
Source: J Fungi (Basel). 2024 Aug 14;10(8):574. doi: 10.3390/jof10080574 (PMC11355149; doi:10.3390/jof10080574)
Supplement: Supplementary file 1 [file jof-10-00574-s001.zip › jof-3019210-supplementary.pdf]

Table S1: Accession numbers of *Fusarium* spp. strains deposited in NCBI GenBank

Sl.. no	Isolate	Accession number	Identity	State	Latitude	Longitude
1.	Raichur	ON385434	*Fusarium verticillioides*	*KA*	16° 09' 31.54'' N	76° 31' 18.26'' E
2.	F4	ON385435	*Fusarium fujikuroi* var *moniliformis*	*MP*	22° 43' 41.0052'' N	74° 33' 38.1096'' E
3.	F7	ON385436	*Fusarium verticillioides*	*RJ*	23° 45' 50.7816'' N	74° 26' 49.5096'' E
4.	F13	ON385437	*Fusarium verticillioides*	*RJ*	24° 5' 4.9524'' N	74° 31' 35.9364'' E
5.	F20	ON385438	*Fusarium verticillioides*	*MP*	22° 36' 21.2292'' N	74° 32' 56.706'' E
6.	F3	ON385439	*Fusarium verticillioides*	*RJ*	23° 28' 33.0348'' N	73° 59' 15.6264'' E
7.	F23	ON385538	*Fusarium verticillioides*	*GJ*	22° 30' 31.5036'' N	73° 22' 42.8016'' E
8.	F17	ON385540	*Fusariumverticillioides*	*RJ*	23° 53' 29.3568'' N	74° 49' 22.116'' E
9.	F34	ON457739	*Fusariumverticillioides*	*RJ*	23° 22' 51.6828'' N	74° 1' 53.688'' E
10.	F47	ON457740	*Fusarium verticillioides*	*GJ*	22° 46' 36.9768'' N	73° 30' 37.0872'' E
11.	FuR15	ON457741	*Fusarium verticillioides*	*RJ*	23°33′41.85″ N	74°28′14.97″ E
12.	F59	OP748382	*Fusarium verticillioides*	*GJ*	22° 53' 26.4948'' N	74° 19' 40.2096'' E
13.	F21	OP748376	*Fusarium verticillioides*	*RJ*	23° 30' 38.8944''N	74° 22' 45.807'' E
14.	FUR10	OP725847	*Fusarium verticillioides*	*RJ*	23°27′43.6″ N	74°22′17.19″ E
15.	W3-2	OP725848	*Fusarium verticillioides*	*TS*	18° 0' 0.1980'' N	79° 35' 17.3940'' E
16.	F12	OP725850	*Fusarium verticillioides*	*RJ*	23° 29' 32.2656'' N	74° 26' 16.1088'' E
17.	F32	OP725846	*Fusarium verticillioides*	*RJ*	23° 39' 53.874'' N	74° 23' 23.0496'' E
18.	B1-1	OP750496	*Fusarium verticillioides*	*KA*	16° 30' 15.5838''N	75° 17' 30.2706'' E
19.	G1-3	OP748378	*Fusarium verticillioides*	*KA*	16 ° 10 '0.1200' 'N	74 ° 49 '59.8728' 'E
20.	Davangere	OP748381	*Fusarium verticillioides*	*KA*	14°31 '49.6452''N	75 ° 48 '3.9384''E
21.	F1	OQ957226	*Fusarium verticillioides*	*RJ*	23° 46' 9.3324'' N	74° 38' 50.9244'' E
22.	FUG 9	OP748380	*Fusarium verticillioides*	*GJ*	23°13′41.45″ N	72°35′17.84 E
23.	F36	OP661212	*Fusarium verticillioides*	*GJ*	22° 39' 57.5172'' N	73° 33' 16.074'' E
24.	FUG2	OQ957227	*Fusarium verticillioides*	*GJ*	23° 5' 49.68'' N	74° 9' 49.97'' E

25.	F6	OP661213	*Fusarium verticillioides*	*RJ*	23° 23' 39.7824'' N	74° 22' 59.8116'' E
26.	FUG 6	OQ957222	*Fusarium verticillioides*	*GJ*	22°26′17.63″ N	73°35′19.64″ E
27.	FUG10	OQ957223	*Fusarium verticillioides*	*GJ*	22° 18' 37.9368'' N	73° 25' 54.1992'' E
28.	F18	OP651068	*Fusarium andiyazi*	*RJ*	23° 15' 13.7016'' N	74° 17' 25.5588'' E
29.	F58	OP661214	*Fusarium andiyazi*	*GJ*	23° 5' 34.7892'' N	74° 11' 21.8508'' E
30.	Mysore	OQ957225	*Fusarium proliferatum*	*KA*	12° 47' 10.4352''N	75° 12' 1.9404''E
31.	F43	OQ957224	*Fusarium proliferatum*	*RJ*	23° 27' 20.7576'' N	74° 24' 58.1868'' E
32.	F49	OQ957221	*Fusarium proliferatum*	*RJ*	23° 44' 45.2832'' N	74° 25' 53.328'' E
33.	F52	OP651067	*Fusarium nygamai*	*RJ*	23° 23' 5.5212'' N	74° 18' 36.0036'' E
34.	F27	OP748379	*Fusarium acutatum*	*RJ*	23° 26' 18.1104'' N	74° 7' 10.8732'' E
35.	F10	OP748383	*Fusarium acutatum*	*RJ*	23° 27' 10.8396''N	74° 20' 54.5244'' E
36.	FUR11	OP725849	*Fusarium acutatum*	*RJ*	23°17′33.6″ N	74° 25′17.19″ E
37.	FUR13	OP748377	*Fusarium acutatum*	*RJ*	23°44′12.23″ N	74°12′14.97″ E
38.	F28	ON385539	*Fusarium acutatum*	*RJ*	23° 36' 0.6192'' N	74° 19' 38.5572'' E
39.	Chokhla	PP827191	Fusarium verticillioides	*RJ*	23° 25' 6.2112'' N	74° 19' 13.8864'' E
40.	F11	PP827192	Fusarium verticillioides	*RJ*	23° 53' 29.3568'' N	74° 49' 22.116'' E
41.	F14	PP827193	Fusarium andiyazi	*RJ*	23° 23' 35.0736'' N	74° 20' 50.5032'' E
42.	F22	PP827194	Fusarium verticillioides	*GJ*	22° 30' 14.148'' N	73° 25' 55.1568'' E
43.	F25	PP827195	Fusarium verticillioides	*RJ*	23° 29' 31.416'' N	74° 23' 21.7104'' E
44.	F27	PP827196	Fusarium acutatum	*RJ*	23° 26' 18.1104'' N	74° 7' 10.8732'' E
45.	F48	PP827197	Fusarium verticillioides	*GJ*	22° 18' 37.9368'' N	73° 25' 54.1992'' E
46.	F8	PP827198	Fusarium verticillioides	*RJ*	23° 45' 42.4116'' N	74° 16' 40.1412'' E
47.	F9	PP827199	Fusarium verticillioides	*RJ*	23° 32' 19.4856'' N	74° 0' 49.5648'' E
48.	Fug4	PP827200	Fusarium verticillioides	*GJ*	22°29′39.38″ N	73°27′54.29″ E
49.	Mandya	PP827201	Fusarium verticillioides	*KA*	12 ° 29 '34.3032' 'N	76 ° 39 '51.4224' 'E
50.	Mandya-2	PP827202	Fusarium verticillioides	*KA*	12° 30' 2.178'' N	76° 54' 20.0628'' E
51.	F19	PP847341	Fusarium verticillioides	*RJ*	23° 28' 41.2536'' N	74° 23' 24.8028'' E
52.	F10	PP847342	Fusarium verticillioides	*RJ*	23° 27' 10.8396'' N	74° 20' 54.5244'' E

53.	F16	PP847343	Fusarium verticillioides	*RJ*	23° 20' 55.1004'' N	74° 18' 35.9928'' E
54.	F2	PP847344	Fusarium verticillioides	*RJ*	23° 33' 54.432'' N	74° 8' 51.8496'' E
55.	F26	PP847345	Fusarium verticillioides	*RJ*	23° 48' 46.7568'' N	74° 37' 49.4724'' E
56.	F31	PP847346	Fusarium verticillioides	*MP*	22° 19' 28.8156'' N	74° 23' 10.392'' E
57.	F33	PP847347	Fusarium verticillioides	*GJ*	22° 47' 42.6228'' N	74° 19' 21.7092'' E
58.	F35	PP850848	Fusarium verticillioides	*RJ*	24° 21' 39.0924'' N	74° 44' 17.8476'' E
59.	F38	PP850849	Fusarium verticillioides	*GJ*	23° 5' 26.16'' N	74° 9' 13.32'' E
60.	F39	PP850850	Fusarium andiyazi	*RJ*	23° 30' 32.0544'' N	74° 24' 16.1316'' E
61.	F42	PP850851	Fusarium verticillioides	*MP*	22° 39' 16.4592'' N	74° 31' 58.6704'' E
62.	F44	PP850852	Fusarium verticillioides	*GJ*	23° 11' 35.6244'' N	73° 53' 14.1576'' E
63.	F45	PP858845	Fusarium verticillioides	*GJ*	23° 5' 21.714'' N	74° 9' 23.3316'' E
64.	F46	PP858846	Fusarium acutatum	*GJ*	22° 58' 57.27'' N	74° 9' 32.6304'' E
65.	F55	PP858847	Fusarium verticillioides	*RJ*	23° 15' 6.0948'' N	74° 10' 30.1908'' E
66.	F57	PP858848	Fusarium verticillioides	*RJ*	23° 10' 32.1168'' N	74° 26' 27.2616'' E
67.	FuG1	PP858849	Fusarium verticillioides	*GJ*	22° 29' 22.2472'' N	73° 36' 27.1425 E
68.	FuG14	PP858850	Fusarium verticillioides	*GJ*	22°32′19.38 “N	73°28′14.23″ E
69.	FuG16	PP862635	Fusarium verticillioides	*GJ*	22°28′ 17.76″ N	73°34′ 2.14″ E
70.	FuG3	PP862636	Fusarium verticillioides	*GJ*	22°23′3.42″ N	73°42′40.98″ E
71.	FuG5	PP862637	Fusarium verticillioides	*GJ*	22°50′19.6″ N	74°4′51.20″ E
72.	FuG7	PP862638	Fusarium verticillioides	*GJ*	22°27′43.94″ N	73°31′0.59 E
73.	FuG8	PP862639	Fusarium verticillioides	*GJ*	22°25′43.94″ N	73°43′22.25″ E
74.	FuR12	PP862640	Fusarium acutatum	*RJ*	23°37′12.23″ N	74°22′14.97″ E

Where RJ-Rajasthan, MP-Madhya Pradesh, GJ-Gujarat, KN-Karnataka, TS-Telangana
